# Magnetic Solid-Phase Extraction of Organic Compounds Based on Graphene Oxide Nanocomposites

**DOI:** 10.3390/molecules25051148

**Published:** 2020-03-04

**Authors:** Natalia Manousi, Erwin Rosenberg, Eleni Deliyanni, George A. Zachariadis, Victoria Samanidou

**Affiliations:** 1 Laboratory of Analytical Chemistry, Department of Chemistry, Aristotle University of Thessaloniki, 54124 Thessaloniki, Greece; 2 Institute of Chemical Technologies and Analytics, Vienna University of Technology, 1060 Vienna, Austria; 3 Laboratory of Chemical and Environmental Technology, Department of Chemistry, Aristotle University of Thessaloniki, 54124 Thessaloniki, Greece

**Keywords:** Graphene oxide, magnetic solid-phase extraction, MSPE, organic pollutants, food samples, environmental samples, biological samples, sample preparation

## Abstract

Graphene oxide (GO) is a chemical compound with a form similar to graphene that consists of one-atom-thick two-dimensional layers of sp^2^-bonded carbon. Graphene oxide exhibits high hydrophilicity and dispersibility. Thus, it is difficult to be separated from aqueous solutions. Therefore, functionalization with magnetic nanoparticles is performed in order to prepare a magnetic GO nanocomposite that combines the sufficient adsorption capacity of graphene oxide and the convenience of magnetic separation. Moreover, the magnetic material can be further functionalized with different groups to prevent aggregation and extends its potential application. Until today, a plethora of magnetic GO hybrid materials have been synthesized and successfully employed for the magnetic solid-phase extraction of organic compounds from environmental, agricultural, biological, and food samples. The developed GO nanocomposites exhibit satisfactory stability in aqueous solutions, as well as sufficient surface area. Thus, they are considered as an alternative to conventional sorbents by enriching the analytical toolbox for the analysis of trace organic compounds.

## 1. Introduction

Solid-phase extraction (SPE) and liquid-liquid extraction (LLE) are two widely used and well-established techniques for the extraction of organic compounds. However, these conventional techniques tend to have many fundamental drawbacks such as complicated and time-consuming steps, requirement for a large amount of organic solvents and sample, as well as difficulties in automation [1,2,3,4]. Recent trends in sample preparation are focused on the progressive replacement of those techniques by miniaturized and environment-friendly techniques, such as solid-phase microextraction (SPME) [5], dispersive liquid–liquid microextraction (DLLME) [6], fabric phase sorptive extraction (FPSE) [7] and dispersive solid-phase extraction (d-SPE) [8].

Magnetic solid-phase extraction (MSPE) is a form of dispersive solid-phase extraction in which a magnetic sorbent is added into an aqueous sample in order to adsorb the target analytes. The sorbent is easily separated by applying an external magnetic field [9]. Subsequently, the analytes are eluted with the addition of an appropriate solvent and magnetic separation is performed again to collect the liquid phase, which is further analyzed. Compared with traditional SPE procedure, with magnetic sorbents there is no need to be packed into SPE cartridges, thus minimizing problems of column blocking and high pressure that are often observed in SPE. Meanwhile, the phase separation with an external magnetic field is a simple and rapid process compared to centrifugation and filtration steps. Sample and organic solvent consumption are also significantly decreased compared to classical SPE and LLE techniques [9,10].

Because of the evolution of technology and nanotechnology, novel extraction sorbents with improved chemical and physical properties have been synthesized and successfully used for magnetic solid-phase extraction of target analytes. Moreover, with the use of these materials, high extraction efficiency, good reproducibility in combination with low detection and quantification limits can be achieved [1,2]. Typical examples of MSPE sorbents are magnetic nanoparticles with surface modification by octadecyl (C_18_) [11], activated carbon [12], carbon-nanotubes [13], graphene [14], graphene oxide [15], metal-organic frameworks [16], covalent organic frameworks [17] and zeolitic imidazole frameworks [18].

Graphene oxide is the oxidized form of graphene that can be easily prepared from natural graphite powder with Hummer’s method after reaction with an anhydrous mixture of sulfuric acid, sodium nitrate and potassium permanganate [19,20,21,22,23,24]. Due to its superior properties such as good thermal and mechanical stability as well as its high surface area, graphene oxide has been used in multiple scientific fields including heterogenous catalysis, gas sorption, storage and separation, sensors and drug delivery [25].

In analytical chemistry, GO has been successfully employed for the sample preparation of a wide variety of samples including biological, food and environmental matrices [26,27,28]. Graphene oxide consists of one-atom-thick two-dimensional layers of sp^2^-bonded carbon and the material is rich in oxygen-containing groups including hydroxyl, carboxyl and epoxy groups, which assist the interaction between the sorbent and organic molecules through strong π-π stacking, hydrophobic interaction and hydrogen bonding [29,30,31].

Graphene oxide is an ultra-light material that poses high dispersibility in aqueous solutions as well as high hydrophilicity which makes its separation from this kind of solutions difficult. In order to improve the separation, GO can form magnetic nanocomposites with magnetite through electrostatic interaction between the negatively charged graphene oxide sheets and the positively charged surface of Fe_3_O_4_ [32]. The magnetic GO nanocomposites combine the high adsorption capacity of graphene oxide and the convenience of magnetic separation. Moreover, the hybrid material can be functionalized with different groups to prevent aggregation and extends its application [33,34].

Many reviews have been published regarding the applications of graphene and graphene-based sorbents in the field of the sample preparation [35,36,37,38,39]. Herein we aim to discuss the applications of magnetic graphene oxide derived nanomaterials for the extraction and preconcentration of organic compounds from environmental, agricultural, biological and food samples.

## 2. Preparation and Applications of GO for the MSPE of Organic Compounds

### 2.1. Nanocomposites of GO with Fe_3_O_4_ Nanoparticles

**Figure 1:**
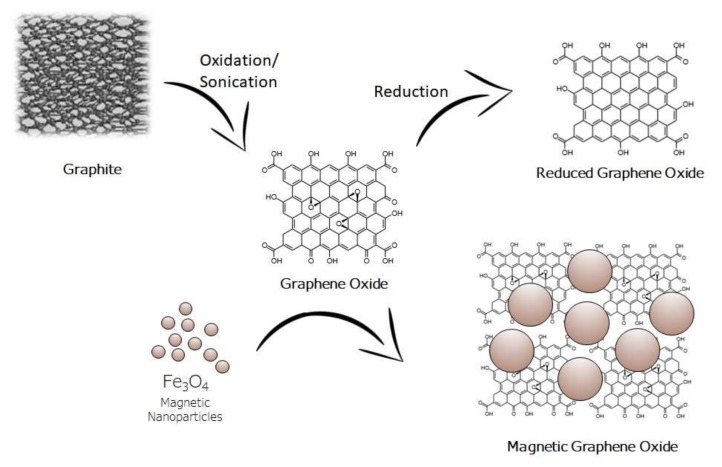
Structure of graphite, graphene oxide, reduced graphene oxide, and magnetic graphene oxide.

Due to its high surface area and its superparamagnetic properties GO/Fe_3_O_4_ has been employed for the extraction of a wide variety of organic compounds from various samples. The surface of magnetic graphene oxide is rich in hydroxyl and carboxyl groups, which assist the interaction between the sorbent and the target analytes through strong π-π stacking, hydrophobic interaction as well as hydrogen bonding [26,27,28,29,30,31]. Figure 1 shows the structure of graphite, graphene oxide, reduced graphene oxide, and magnetic graphene oxide.

The one-step co-precipitation approach is the most common synthetic route for the preparation of magnetic GO. In this approach, graphene oxide is dispersed in water. Subsequently, salts of Fe^2+^ (e.g., ferrous chloride) and of Fe^3+^ (e.g., ferric chloride) are added, the mixture is heated and ammonium hydroxide is slowly added and the magnetite nanoparticles are formed [26].

Another common synthetic procedure is the solvothermal approach, in which GO is added in a dispersion of a Fe^3+^ salt (e.g., ferric chloride hexahydrate) and sodium acetate, under vigorously stirring for 30 min at room temperature [33,40]. The dispersion is transferred in an autoclave and heated under reflux for a specific time span. The ferric salt is added as the iron source, while the sodium acetate assists both the electrostatic stabilization of the magnetic nanoparticles onto GO in order to prevent agglomeration of the particles and the reduction of Fe^3+^ to Fe_3_O_4_.

Magnetic GO can be prepared by mixing GO and Fe_3_O_4_ under ultrasonic irradiation, stirring or mechanical shaking. In this case, Fe_3_O_4_ nanoparticles are dispersed in nitric acid solution in an ultrasonic bath for 30 min in order to generate a positively charged surface. Graphene oxide is dispersed in deionized water in order to generate a negatively charged surface. Subsequently, the dispersions of Fe_3_O_4_ and GO are mixed, the pH of the mixture is adjusted to a desired value and the mixture is subjected to vigorous magnetic stirring, mechanical shaking or ultrasonic radiation for a certain time span (e.g., 5 h) [41]. Finally, the GO/Fe_3_O_4_ material can be prepared after hydroxylation of Fe_3_O_4_ nanoparticles by mixing with GO and dimethyl sulfoxide. After ultrasonic treatment of the mixture for a certain time at room temperature, the desired material is obtained [42].

Organic molecules of various chemical groups have been extracted with GO/Fe_3_O_4_. Table 1 summarizes the applications of GO/Fe_3_O_4_ for the MSPE of organic compounds from biological, environmental and food samples.

Regarding the analysis of biological samples, graphene oxide has been employed for the MSPE of tamsulosin hydrochloride [43], pseudoephedrine [44], psychoactive drugs (including morphine, 6-monoacetylmorphine, amphetamine, methamphetamine, codeine, cocaine, dolantin and benzoylecgonine) [45], methamphetamine [46], flavonoids [33], as well as monohydroxy polycyclic aromatic hydrocarbons [47]. High-performance liquid chromatography (HPLC) with ultraviolet detector (UV), diode array detector (DAD), mass spectrometer (MS), or tandem mass spectrometer (MS/MS) have been employed for the separation and determination of the target analytes. The MSPE methods provide good extraction recoveries (>80%) and low limit of detections (LODs). Since the adsorbents provided nearly quantitative extraction recoveries in most cases, it can be concluded that the differences of the LOD values for the same compounds in similar sample matrices, mainly depend on the detection technique (e.g., DAD, MS, tandem MS, etc.). Moreover, the GO/Fe_3_O_4_ material was found to be reusable in most cases.

**Figure 2:**
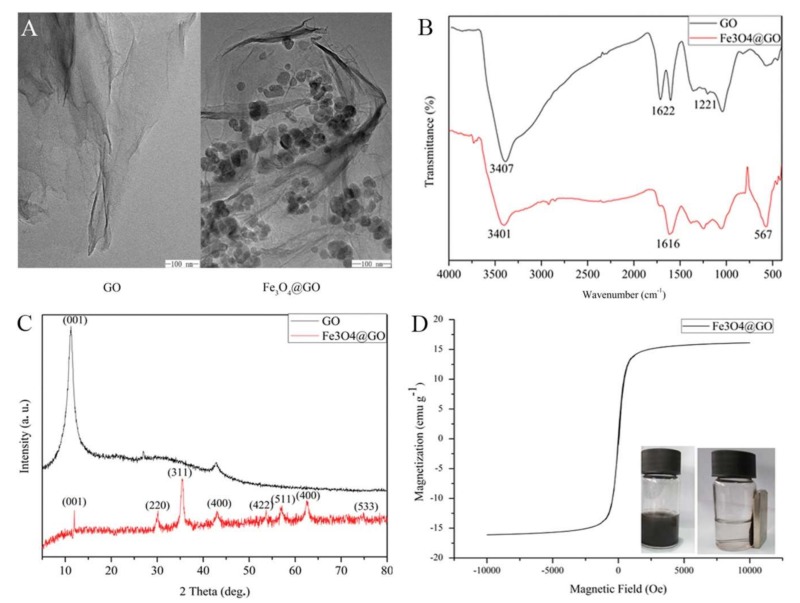
TEM (**a**), FT-IR (**b**), XRD (**c**) and vibrating sample magnetometer (VSM) (**d**) of GO/Fe_3_O_4_. Reproduced with permission from [57]. Copyright Elsevier, 2017.

The GO/Fe_3_O_4_ magnetic nanocomposite has been employed for the extraction of 2,4,4′-trichlorobiphenyl (PCB 28) [34] and atrazine [48] from water samples prior to their determination by gas chromatography-mass spectrometry (GC-MS). Furthermore, di-2-ethylhexyl phthalate (DEHP) [49], polycyclic aromatic hydrocarbons (PAHs) [32], 2,4,6-trinitrotoluene (TNT) [41], malachite green and crystal violet [50], the cytostatic drugs imatinib and doxorubicin [51] as well as sulfonamides [52] have been extracted from water samples prior to their separation with HPLC and determination using a UV or DAD detector. Figure 2 shows the transmission electron microscopy (TEM) image, (a), a Fourier-Transformation Infrared spectroscopy (FT-IR) image (b), an X-ray diffraction (XRD) image (c) and a vibrating sample magnetometer (VSM) image (d) of GO/Fe_3_O_4_.

Sulfonamides have been extracted from milk samples by GO/Fe_3_O_4_ sorbent prior to their determination by HPLC–MS/MS [53] or capillary electrophoresis-diode array detection (CE-DAD) [54]. Sulfadiazine has been extracted from milk, honey and water samples with GO/Fe_3_O_4_ prior to its spectrophotometric and optimized angle mode-mismatched thermal lens spectrometric determination (OAMTLS) [55].

Other applications of GO/Fe_3_O_4_ for the extraction of organic compounds from food samples include the extraction of patulin from apple juice [56], flavors and fragrances (including ethyl vanillin, trans-cinnamic acid, methyl cinnamate, ethyl cinnamate, and benzyl cinnamate) from orange juice, chocolate and fruit sugar [57], lignans from sesame oil [42], flavonoids from tea, wine and urine samples [33], azo dyes (tartrazine, amaranth, carmine, sunset yellow, allura red) from jellies and candies [40].

The organic solvents that were mainly used for the elution of the adsorbed analytes from the GO/Fe_3_O_4_ nanomaterial include acetone, n-hexane, acetonitrile, methanol etc., however, only a small quantity of these solvents was required. In most cases, the GO/Fe_3_O_4_ sorbent was found to be reusable after a regeneration and a washing step. The as-prepared nanomaterial has some significant benefits including high surface area, superparamagnetic properties and strong magnetism. The synthetic procedures that were reported were simple, mild, rapid an economic and the MSPE method provided the benefits of limited organic waste, no requirement for time-consuming centrifugation, column-passing or filtration steps.

### 2.2. Nanocomposites of Reduced GO with Fe_3_O_4_ Nanoparticles

Reduced graphene oxide (RGO) is a nanomaterial obtained by chemical reduction of graphene oxide that contains less oxygen groups and has properties closer to those of graphene [25,58]. RGO has various applications such as removal of metals and dyes [59,60], catalysts [61], electroanalytical sensors [62] etc. Magnetic nanocomposites of reduced graphene oxide have been successfully applied for the MSPE of various analytes from different sample matrices. Due to the combination of the magnetic Fe_3_O_4_ nanoparticles and the graphene sheets, the magnetic RGO sorbent shows distinguished properties including good dispersity, high surface area, high adsorption efficiency and good super-paramagnetism [63].

**Figure 3:**
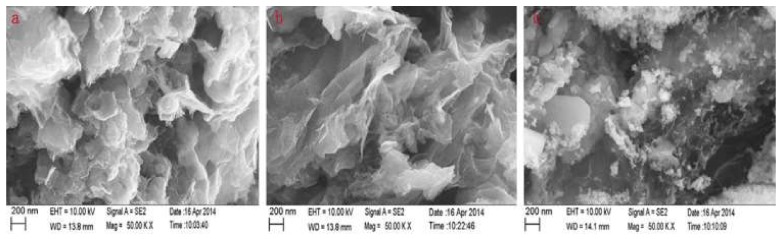
Scanning electron micrographs of reduced graphene oxide (RGO)/Fe_3_O_4_ prepared by solvothermal (**a**), hydrothermal (**b**) and co-precipitation (**c**) methods. Reproduced with permission from [64]. Copyright Elsevier, 2015.

There are different synthetic procedures for the fabrication of RGO/Fe_3_O_4_ sorbents. The co-precipitation approach is a multi-step procedure in which GO/Fe_3_O_4_ previously prepared by precipitating Fe^2+^ and Fe^3+^ in the presence of GO is reduced with the addition of hydrazine hydrate [64,65]. For the solvothermal approach, graphite oxide is exfoliated in diethylene glycol under sonication to produce graphene oxide while ferric chloride with sodium acetate are also dissolved in diethylene glycol. In this case, diethylene glycol was both solvent and reducing agent. Accordingly, the GO dispersion is added into the second solution and the mixture was sonicated and heated at 190 °C in an autoclave [64,66]. The RGO/Fe_3_O_4_ can be also prepared through the hydrothermal method, which is similar to the solvothermal but instead of organic solvents that are used in the solvothermal method, water is used as a solvent in the hydrothermal approach. For this purpose, a salt of Fe^3+^ and sodium hydroxide is added to an aqueous solution of graphene oxide and the mixture is heated in an autoclave for a certain time span [64,67]. Figure 3 shows the scanning electron micrographs (SEM) of RGO/Fe_3_O_4_ prepared by solvothermal *(a)*, hydrothermal *(b)* and co-precipitation *(c)* methods.

Sudan dyes have been extracted from tomato sauce and chili-containing foods with RGO/Fe_3_O_4_ prior to their determination by HPLC-DAD [68]. The developed MSPE procedure was simple, economic and provided satisfactory extraction recoveries and LOD values. Magnetic RGO/Fe_3_O_4_ has been also used for the MSPE of bisphenol A from water samples prior to its determination by HPLC-UV. The MSPE technique was coupled with dispersive liquid–liquid microextraction (DLLME) in order to utilize the benefits of both sample preparation techniques. The magnetic sorbent was separated conveniently and rapidly from the sample matrix and it was found to be reusable for at least 12 repeated cycles [69].

In 2016, Mehdinia et al. developed a microwave-assisted synthesis of reduced graphene oxide decorated with magnetite and gold nanoparticles. Gold nanoparticles offer the benefits of chemical stability, biocompatibility, satisfactory magnetic properties and possibility for chemical modification. The RGO/Fe_3_O_4_@Au sorbent was used for the MSPE of organochlorine pesticides from seawater samples prior to their determination with GC-MS [70].

A magnetic polyethyleneimine functionalized reduced graphene oxide nanocomposite was synthesized and used for the MSPE of polar non-steroidal anti-inflammatory drugs from water samples [71] and polar acidic herbicides from rice [72]. The modification of RGO with polyethyleneimine changed the polarity of RGO to some extent and offered more active sites for the adsorption of the polar target analytes.

In 2018, Feng et al. synthesized RGO/Fe_3_O_4_-carbon nanotubes composite via a simple and green one-pot solvothermal approach. For this purpose, iron (III) chloride hexahydrate, sodium acetate, GO and CNTs were added in a Teflon-liner and the suspension was heated at 200 °C for 10 h. It was found that the Fe_3_O_4_ nanoparticles were covered with RGO and CNTs, while CNTs were inserted between the RGO sheets in order to prevent the aggregation of the RGO sheets. The novel sorbent was used for the MSPE of sulfonamides from milk prior to their determination with HPLC-UV [73]. Pinsrithong and Bunkoed prepared a hierarchical porous polypyrrole-coated nanostructured composite of RGO and Fe_3_O_4_ nanoparticles. After incorporation into alginate hydrogel microspheres, the developed sorbent was successfully applied for the MSPE of phthalates from bottled drinks [74].

In order to increase the selectivity of the conventional RGO/Fe_3_O_4_ sorbent, Mahpishanian and Sereshti reported the one-step green synthesis of β-cyclodextrin/iron oxide-RGO nanocomposite. The sorbent was used for the MSPE organochlorine pesticides residue from honey samples prior to their determination by gas chromatography-electron capture detection (GC-ECD). In this case, functionalization with β-cyclodextrin was chosen, due to the high potential supramolecular recognition capability of β-cyclodextrin [75].

Akbarzade et al. synthesized zero valent Fe-RGO quantum dots and used them for the MSPE of organophosphorus pesticides from real water and fruit juice samples prior to their determination with GC-MS. The quantum dots were prepared with a hydrothermal cutting method and sodium borohydride was used for the reduction. The novel sorbent provided short extraction time, simple operation as well as high enhancement factor [76]. 

### 2.3. Functionalized Nanocomposites of GO with Fe_3_O_4_ Nanoparticles

The main disadvantage of graphene oxide-based adsorbents is the important π–π stacking interactions between graphene oxide nanosheets, which are responsible for serious aggregation and restacking of the nanosheets, resulting in a potential block of the active adsorption sites of the sorbent and a decrease of its specific surface area. In order to overcome this problem, functionalization of the sorbent with different molecules that can enter between the GO nanosheet and prevent them from aggregation and restacking can take place [77].

In order to overcome this limitation, Yilmaz et al. developed a magnetic nanodiamond/graphene oxide hybrid and used it for the MSPE of sildenafil from alleged herbal aphrodisiacs by HPLC-DAD system. Functionalization with nanodiamond successfully prevented the aggregation and restacking of GO nanosheets [77].

Functionalization of graphene oxide-based adsorbents can also take place to enhance the extraction efficiency of the material by introducing compatible chemical molecules with high surface area and abundant functional groups in the structure of GO.

Polyamidoamine (PAMAM) dendrimer has been used to develop amino-terminated hyper-branched PAMAM polymer grafted magnetic graphene oxide nanosheets for the MSPE of selective serotonin reuptake inhibitors from plasma samples [78]. Due to the large number of terminal groups of polyamidoamine dendrimer, the structural characteristics as well as the internal spaces between their branches, which can trap the target analyte, the functionalized GO/Fe_3_O_4_ sorbent exhibited higher extraction efficiency compared to the conventional GO/Fe_3_O_4_ nanocomposite.

Functionalization with soluble eggshell membrane protein (SEP) was found to increase the stability and adsorption performance as well as accuracy and recoveries of GO/Fe_3_O_4_ due to the high density of surface functional groups such as amines, amides and carboxylic groups of SEP [79].

Porphyrin has been also used for the functionalization of GO/Fe_3_O_4_ nanocomposite and the sorbent was used for the MSPE of sulfonamides from tap and river water samples [80]. Due to the π-π stacking and electrostatic attraction between the negatively charged functionalized nanocomposite and the positively charged sulfonamides, the extraction process was accelerated. The novel sorbent showed higher adsorption capacity than the conventional GO/Fe_3_O_4_.

A co-polymer of divinylbenzene (DVB) and glycidylmethacrylate (GMA) was used for the functionalization of GO/Fe_3_O_4_ in order to develop a sorbent for the MSPE of chlorophenols from environmental water prior to their determination by HPLC-MS/MS [81]. Due to π-π stacking and hydrogen-bonding interactions between the analytes and the functionalized adsorbent, good extraction efficiency was observed.

Polystyrene (PS) [82] and poly(pyrrole-co-aniline) [83] are two examples of functional groups that were employed to prepare magnetic graphene functionalized nanocomposites, which were used for the MSPE of PAHs from water samples. Polystyrene is rich in phenyl and alkyl groups. Therefore, functionalization with PS enhanced the extraction efficiency by increasing the active surface sites of the material. The sorbent exhibited sufficient surface area, excellent magnetic properties and resulted in good extraction efficiencies and low detection limits [82]. Similarly, the poly(pyrrole-co-aniline) functionalized graphene oxide nanocomposite combined the properties of the polypyrrole and polyaniline co-polymer, the GO, and the magnetic nanoparticles. As a result, the developed nanocomposite exhibited a significant enhancement of extraction efficiency due to the increased number of active surface sites on the sorbent as well as the protection of the Fe_3_O_4_ nanoparticles [83].

Polythionine was also employed for the functionalization of magnetic graphene oxide through an oxidative polymerization reaction of thionine on the surface of GO/Fe_3_O_4_ [84,85]. This surface modification significantly improved the merits of GO/Fe_3_O_4_, providing satisfactory extraction efficiency. The functionalized nanocomposite was used for the MSPE of chlorpheniramine [84] and duloxetine [85] from human plasma prior to their determination by HPLC-UV.

In order to enhance the dispersibility of magnetic GO in hydrophobic media, functionalization with phytic acid has been reported. Phytic acid-stabilized GO/Fe_3_O_4_ was applied for extraction of PAHs from vegetable oils. Due to the super-amphiphilicity of phytic acid, the dispersibility of the conventional GO/Fe_3_O_4_ sorbent increased. [86].

Functionalization of GO/Fe_3_O_4_ can also be performed for the enhancement of its selectivity towards the target analytes. In 2015, Abdolmohammad-Zadeh and Talleb synthesized a β-cyclodextrin (β-CD) grafted GO/Fe_3_O_4_ nano-hybrid and used it for the MSPE of gemfibrozil from human serum and pharmaceutical waste-water samples followed by determination using spectrofluorometry. This chemical compound can selectively bind with various organic, inorganic and biological guest molecules into its cavity to form stable host–guest inclusion complexes by a series of forces such as hydrophobic and van der Waals interactions. Therefore, due to the surface modification of graphene oxide with β-cyclodextrin, selective separation of the target analyte from complex sample matrices was achieved [87].

Other examples of chemical molecules that have been used for the functionalization of GO/Fe_3_O_4_ are triethylenetetramine [88], silica [89], sporopollenin [90] and phenylethyl amine [91]. The superior adsorption capacity of the functional groups resulted in functionalized magnetic GO nanocomposites with sufficient surface area, excellent extraction efficiency, good stability as well as ease in handling and separation. Table 2 summarizes the application of functionalized GO/Fe_3_O_4_ nanocomposites. 

**Figure 4:**
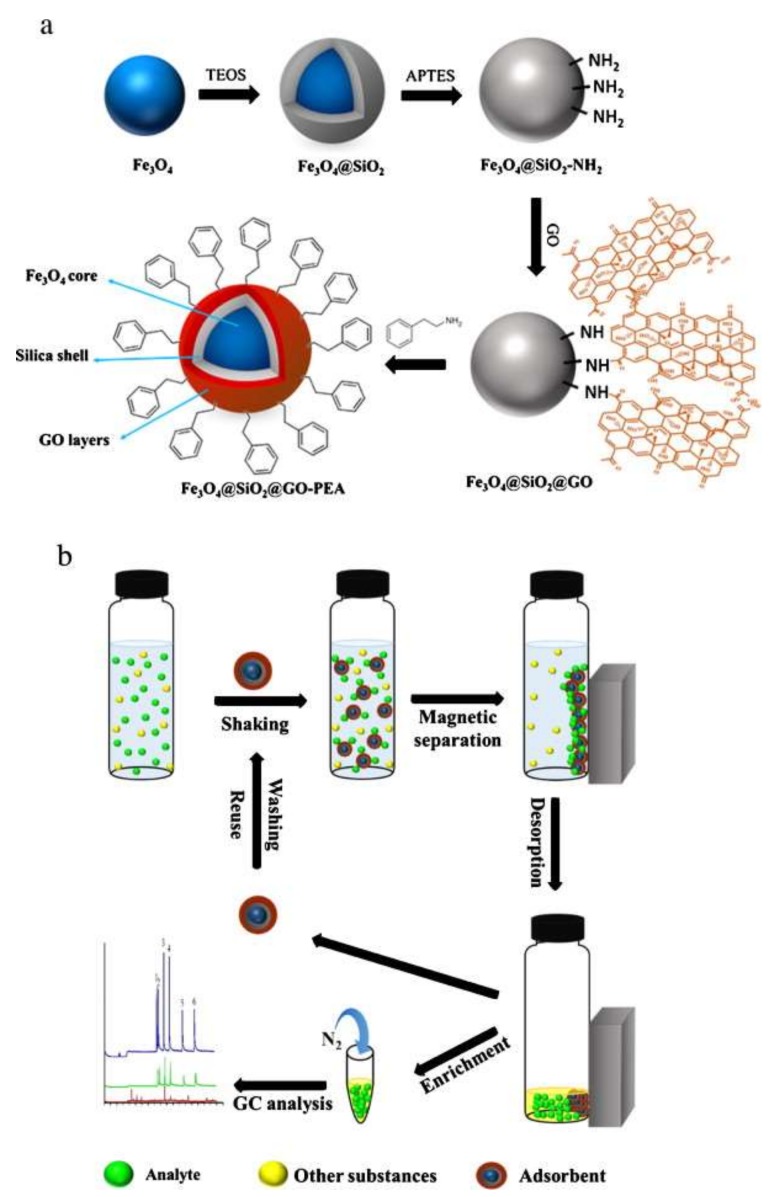
Synthesis of phenylethyl amine functionalized GO/Fe_3_O_4_ (**a**) and its application on the MSPE procedure (**b**). Reproduced with permission from [91]. Copyright Elsevier, 2015.

Moreover, Figure 4 summarizes the synthesis of phenylethyl amine functionalized GO/Fe_3_O_4_ and its application in the MSPE procedure.

### 2.4. Functionalized Nanocomposites of Magnetic GO with MIPs

Molecularly imprinted polymers (MIPs) are highly selective, tailor-made synthetic polymeric materials that exhibit high adsorption capacity. Moreover, they can be easily prepared with economic synthetic procedures. Therefore, MIPs have been applied for the extraction and preconcentration of trace analytes in diverse fields, including natural, agricultural, and food products and environmental samples [92,93,94]. The combination of graphene oxide and molecularly imprinted polymers can significantly enhance the selectivity of the extraction procedure [93,94].

Ning et al. developed a molecularly imprinted polymer on magnetic GO and used it for the extraction of 17β-estradiol from milk powder samples. For this purpose, GO/Fe_3_O_4_ nanoparticles were grafted with acrylic acid. Subsequently, the MIPs-GO/Fe_3_O_4_ sorbent was prepared from 17β-E2 (template molecule), acrylamide (functional monomer), ethylene glycol dimethacrylate (cross-linker), and 2,2′-azobis(isobutyronitrile) (initiator) in acetonitrile dispersion of the functionalized magnetic GO material. With the use of developed sorbent, specific recognition, as well as highly effective removal of 17β-E2 from complicated matrices, were achieved [95]. Barati et al. synthesized a molecular imprinted polymer based on magnetic chitosan/graphene oxide and used it for the selective extraction of fluoxetine from environmental and biological samples prior to its spectrophotometric determination. Due to the multi imprinting sites and high surface area of the magnetic chitosan/GO, high selectivity and adsorption efficiency was observed [96].

### 2.5. Functionalized Nanocomposites of Magnetic GO with MOFs

Metal-organic frameworks are mixed organic-inorganic supramolecular materials that became popular in 1995, when Yaghi and Li reported the synthesis of a MOF with large rectangular channels [97]. These materials are based on the coordination of metal ions or clusters with bi- or multidentate organic linkers [98,99]. Metal-organic frameworks exhibit various extraordinary properties including luminosity, tunable pore size, flexibility and thermal stability as well as high surface areas [100,101].

The combination of graphene oxide and metal organic frameworks enhances the merits of sorbent including its reusability, its pore volume, its dispersion capability, its extraction capacity, its mechanical strength as well as its surface area [102,103,104].

Liu et al. developed a sorbent based on magnetic graphene oxide functionalized MOF-199 with the aim to combine the benefits of GO/Fe_3_O_4_ and MOFs for the rapid separation and highly selective adsorption of triazole pesticides [105]. The sorbent was employed for the MSPE of triazole pesticides from environmental water samples prior to their determination by HPLC-MS/ΜS. High adsorption capacity for the target analytes was observed due to the high surface area and pore volume of the novel nanocomposite.

A high-affinity graphene oxide-encapsulated magnetic zirconium-MOF was developed for the MSPE of photosensitizers hematoporphyrin and hematoporphyrin monomethyl ether from human urine prior to their determination by ultra-performance liquid chromatography-high resolution mass spectrometry (UPLC-HRMS) [106]. The novel sorbent combined the advantages of GO, magnetic nanoparticles with the advantages of large surface area, high porosity, and easy modification of metal-organic frameworks.

**Figure 5:**
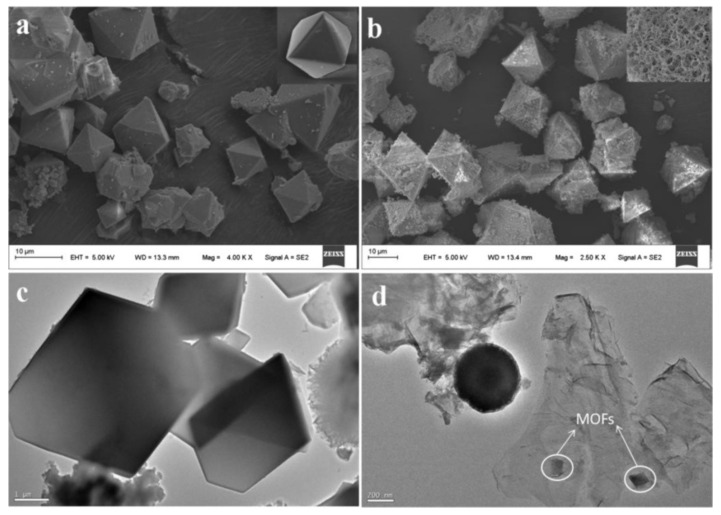
SEM images of Cu-MOFs (**a**) and Fe_3_O_4_@SiO_2_-GO-MOFs (**b**) and TEM images of Cu-MOFs (**c**) and Fe_3_O_4_@SiO_2_-GO-MOFs (**d**). Reproduced with permission from [107]. Copyright Elsevier, 2018.

Wang et al. synthesized magnetic Cu-MOFs embedded within graphene oxide nanocomposites and used it for the MSPE of benzenoid-containing insecticides prior to their determination by HPLC-UV [107]. For this purpose, GO nanosheets were functionalized with silica-coated Fe_3_O_4_ nanoparticles with core-shell structured through covalent bonding and subsequently the GO surfaces were modified with Cu-MOFs. The silica shells prevented the oxidation and agglomeration of Fe_3_O_4_ nanoparticles and served as a platform to integrate the Fe_3_O_4_ particles and GO nanosheets through covalent bonds. Finally, the functionalization with the Cu-MOFs enhanced the extraction efficiency by providing more active sites for adsorption because of the high porosity and tunability of MOFs. Figure 5 shows the SEM images of TEM Cu-MOFs (a) and Fe_3_O_4_@SiO_2_-GO-MOFs (b) as well as the TEM Cu-MOFs (c) and Fe_3_O_4_@SiO_2_-GO-MOFs (d).

Pourbahman et al. synthesized a graphene oxide/metal–organic framework-74/Fe_3_O_4_/polytyramine (GO/MOF-74/Fe_3_O_4_/PTy) nanoporous composite and used it for the magnetic dispersive micro solid-phase extraction (MD-μ-SPE) of prokinetic drugs prior to their determination with HPLC–UV [102]. The surface modifiers were employed to improve the properties of graphene oxide, including the surface area-to-volume ratio, the adsorption capacity as well as its selectivity. Other nanocomposites of GO and metal-organic frameworks were reported by the working group of Cao [103] and the working group of Zhang [104]. The first group synthesized a magnetic zeolitic imidazolate framework (ZIF) 67/graphene oxide composite material and used it for the MSPE of neonicotinoid insecticides from environmental water samples [103]. The combination of the ZIF and GO nanoparticles was performed to facilitate enhancement of the sorbent properties. The second group synthesized a magnetic zeolitic imidazolate framework-7 supported graphene oxide nanocomposite with the assistance of polydopamine and used it for the MSPE of fungicides from environmental water and soil samples. It was found that the combination of ZIF units and GO layers synergistically enhanced the extraction of fungicides [104]. However, in both cases, no information on sorbent reusability was provided.

### 2.6. Functionalized Nanocomposites of Magnetic GO with MOPs

Microporous organic polymers (MOPs) represent a class of amorphous porous materials, composed of fully covalently bound organic building blocks. MOPs have lately gained much research interest, due to the combined superiority of porous materials and functional polymers [108,109,110]. MOPs exhibit well-defined porosity as well as high surface area. Because of their tunable surface chemistry, MOPs can be easily functionalized [108]. Depending on the choice of monomers, functionality and polymerization method, MOPs can be prepared both as solution processable or as insoluble networked materials [109]. Therefore, MOPs have various applications including gas storage [111], environmental remediation [112], catalysis [113], energy storage [114] etc. In analytical chemistry, MOPs have been used as sorbents for the extraction of different organic compounds including hydroxylated PAHs [115] and 5-nitroimidazoles [116].

By combining MOPs and magnetic GO it is possible to develop nanocomposites that combine the extraordinary properties of both MOPs and GO. Shahrebabak et al. synthesized a triazine-based polymeric network modified magnetic nanoparticles/GO nanocomposite and used it for the MSPE of basic and acidic pesticides from food (e.g., cucumber, tomato) and water samples by HPLC-UV. For the fabrication of the material, Fe_3_O_4_ nanopaticles were modified by triazine-based polymeric prepared from melamine and terephthaldehyde. Subsequently, the functionalized Fe_3_O_4_ nanoparticles were mixed with GO in THF and the mixture was sonicated. The novel sorbent showed well-defined porosity, high surface area, good chemical stability and tunable surface chemistry. Due to the different functional groups of the nanocomposite (i.e., amine and carboxylic groups), simultaneous extraction of basic and acidic pesticides was achieved [108].

### 2.7. Applications of Magnetic GO Nanocomposites Modified with Ionic Liquids (ILs) and Deep Eutectic Solvents (DESs)

Ionic liquids (ILs) and deep eutectic solvents (DESs) are an alternative to environmentally harmful ordinary organic solvents. ILs are generally composed of bulky, non-symmetrical organic cations (i.e., imidazolium, ammonium pyrrolidinium, pyridinium etc.) and different inorganic or organic anions [117,118,119]. DESs are systems formed from a eutectic mixture of Lewis or Brønsted acids and bases that contain a variety of anionic and/or cationic species [120,121]. ILs and DESs have a tunable nature and their properties can be optimized through the choice of their cationic and anionic constituents [118,119]. Although DESs and ILs have similar physical properties, their chemical properties differ resulting in different potential applications [122,123]. By combining ionic liquids and deep eutectic sorbents with magnetic graphene oxide it is possible to design and develop new extraction sorbents with extraordinary properties [117,123].

In 2016, Cai et al. synthesized a planar graphene oxide-based magnetic ionic liquid nanomaterial for extraction of chlorophenols from environmental water samples coupled with HPLC-MS/MS [123]. For this purpose, Fe_3_O_4_@SiO_2_ magnetite microspheres functionalized with amino-groups (Fe_3_O_4_ @SiO_2_-NH_2_) and 1-carboxymethyl-3-methylimidazolium chloride were used to functionalize graphene oxide. The novel sorbent exhibited great adsorption capacity and was successfully used to extract both polar and non-polar chlorophenols from tap, river and well water. The sorbent was found to be reusable for at least six times.

Wu et al. developed a mixed hemimicelles MSPE method for the extraction of cephalosporins from biological samples based on ionic liquid-coated magnetic graphene oxide nanoparticles prior to their determination by HPLC-UV [124]. For the fabrication of the modified sorbent, GO/Fe_3_O_4_ nanoparticles were treated with 1-hexadecyl-3-methylimidazoliumbromide in a phosphate buffer solution (pH 7.0) and the mixture was subjected to ultrasonic mixing 5 min. Due to the π-π stacking, the hydrophobic and the electrostatic interactions between the mixed hemimicelles and analytes, the modified material exhibited high surface area and excellent adsorption capacity. Moreover, after the extraction and desorption cycle, it is possible to regenerate the GO/Fe_3_O_4_ with water and methanol. A decrease in the sorbent amount was observed after six cycles, however no significant loss of the sorption capacity occurred.

A three-dimensional ionic liquid functionalized magnetic GO/Fe_3_O_4_ nanocomposite was prepared and used for the MSPE of PAHs from vegetable oil prior to their determination by GC–MS [125]. The ionic-liquid modified sorbent was prepared with a one-pot solvothermal method with 1-(3-aminopropyl)-3-methylimidazolium bromide. Subsequently, free-radical copolymerization took place to form a porous and 3D structure. After modification, the modified material exhibited high adsorption toward PAHs due to strong π-π bonds between the sorbent and the analytes, as well as good mechanical stability and excellent reusability (for at least five times).

Lamei et al. evaluated different DESs, based on choline chloride (ChCl) for the modification of GO/Fe_3_O_4_, in order to develop a MSPE sorbent for the preconcentration of methadone in biological and water samples followed by GC–FID and GC–MS [126]. For this purpose, choline chloride was used as hydrogen bond acceptor and phenol, glycerol, and 5,6,7,8-tetrahydro-5,5,8,8-tetramethylnaphthalen-2-ol (TNO) as hydrogen-bond donors. The modified nanocomposites were prepared by mixing the DES and the GO/Fe_3_O_4_ in a round-bottomed flask under stirring at room temperature for 1 h. The highest extraction recovery for methadone was observed with the TNO hydrogen-bond donors at a molar ratio of 1:2. of ChCl:TNO. The developed methods exhibited high preconcentration factor and satisfactory extraction recovery, however no reusability data were reported.

## 3. Conclusions

Due to the sufficient surface area and its superparamagnetic properties, magnetic graphene oxide has successfully been employed for the extraction of a wide variety of organic compounds from environmental, food, biological and agricultural samples. The surface of magnetic GO/Fe_3_O_4_ nanocomposite contains a large amount of hydroxyl and carboxyl groups, which assist the interaction between the sorbent and organic molecules through π-π stacking, hydrophobic interaction as well as hydrogen bonding. In most MSPE methods, the GO/Fe_3_O_4_ sorbent was found to be reusable after the desorption step, indicating satisfactory stability of the materials. Moreover, the synthetic procedures for the preparation of magnetic graphene oxide are simple, mild, rapid and economic.

The main limitation of graphene oxide-based adsorbents is aggregation and restacking of the nanosheets, which may lead to blocking of the active adsorption sites of the sorbent, as well as to the reduction of its specific surface area. A wide variety of functional groups have been employed to overcome this problem. Enhancement of the dispersibility of magnetic graphene oxide in hydrophobic media can be also achieved through functionalization with appropriate chemical groups. Functionalization of magnetic graphene oxide has been also employed in order to enhance the extraction efficiency of the nanocomposite by introducing compatible chemical molecules with high surface area. Moreover, by using functional groups that can selectively bind with various organic, inorganic and biological guest molecules such as β-cyclodextrin, the selectivity of the extraction towards the target analyte can be significantly improved.

Microporous organic polymers, molecular imprinted polymers, metal-organic frameworks, and zeolitic imidazole frameworks have been used in order to create nanocomposites with extraordinary properties. The resulted materials combined the advantages of magnetic graphene oxide and the chosen material and exhibited sufficient surface area, great extraction efficiency, ease in handling and separation and good stability.

Finally, modification of magnetic graphene oxide with ionic liquid and deep eutectic solvents can significantly enhance the extraction efficiency of the sorbent. In this case, the modification process is simple, rapid, and environmentally friendly since no toxic organic solvents are required. Moreover, magnetic procedures are energy and time saving, and they require only small quantities of solvents during extraction and elution steps; therefore, they play a key role in green analytical chemistry.

## Figures and Tables

**Table 1:** Applications of GO/Fe_3_O_4_ for the magnetic solid-phase extraction (MSPE) of organic compounds from biological, environmental and food samples.

Analyte	Sample Matrix	Synthetic Route	Analytical Technique	LODs (ng mL^−1^)	Recovery (%)	Reusability	Reference
*Biological Samples*							
Tamsulosin hydrochloride	Plasma	Mix/Stirring	HPLC-UV	0.17	98.1–101.4	At least 4 times	[43]
Pseudoephedrine	Urine	Chemical coprecipitation	HPLC-UV	25	96.42	N.A.	[44]
Psychoactive drugs	Urine	Chemical coprecipitation	UHPLC-MS/MS	0.02–0.2	80.4–105.5	At least 10 times	[45]
Methamphetamine	Urine	Chemical coprecipitation	HPLC-UV	30	93.5	At least 1 time	[46]
PAH metabolites	Urine	Mix/Agitation	UHPLC-MS	0.01–0.15	98.3–125.2	N.A.	[47]
*Environmental Samples*							
PCB 28	Water	Chemical coprecipitation	GC-MS	0.027–0.059	77.2–99.7	N.A.	[34]
Atrazine	Water	Chemical coprecipitation	GC-MS	0.6 × 10^−3^	96–102	N.A.	[48]
DEHP	Water	Solvothermal approach	HPLC-DAD	0.35	91.6–106.5	N.A.	[49]
PAHs	Water	Chemical coprecipitation	HPLC-UV	0.09–0.19	76.8–101.2	N.A.	[32]
TNT	Water	Mix/Stirring	HPLC-UV	0.3	87–120	Up to 6 times	[41]
Malachite Green, Crystal Violet	Water	Chemical coprecipitation	HPLC-UV	0.091–0.12	91.5–116	N.A.	[50]
Imatinib, doxorubicin	Water	Mix/Stirring	HPLC-UV	1.8–1.9	88.4–96.7	N.A.	[51]
Sulfonamides	Water	Chemical coprecipitation	HPLC-DAD	50–100	67.4–119.9	N.A.	[52]
*Food Samples*							
Sulfonamides	Milk	Chemical coprecipitation	HPLC-MS/MS	0.02–0.13	73.4–97.4	At least 6 times	[53]
Sulfonamides	Milk	Solvothermal approach	CE-DAD	0.89–2.31	62.7–104.8	At least 3 times	[54]
Sulfadiazine	Milk, honey, water	Chemical coprecipitation	Spectrophotometry	340	94.3–100.7	At least 10 times	[55]
Patulin	Apple juice	Chemical coprecipitation	HPLC-UV	2.3 ng g^−1^	68.7–83.6	At least 10 times	[56]
Flavors, fragrances	Orange juice, chocolate, fruit sugar	Chemical coprecipitation	HPLC-DAD	20–40	71.5–112.4	At least 5 times	[57]
Flavonoids	Tea, wine, urine	Solvothermal approach	HPLC-DAD	0.2–6.0	82.0–101.4	N.A.	[33]
Azo dyes	Jelly, candy, plum candy	Solvothermal approach	HPLC-UV	0.36–2.23 ng g^−1^	73.2–107.7	Up to 6 times	[40]
Lignans	Sesame oil	Hydroxylation, sonication	HPLC-UV	20–50 ng g^−1^	84.6–86.8	N.A.	[42]

**Table 2:** Application of functionalized GO/Fe_3_O_4_ nanocomposites for the MSPE of organic compounds.

Analyte	Sample Matrix	Functional Group	Analytical Technique	LODs (ng mL^−1^)	Recovery (%)	Reusability	Reference
*Biological Samples*							
Chlorpheniramine	Plasma	Polythionine	HPLC-UV	0.4	87.9–96.4	At least 6 times	[84]
Duloxetine	Plasma	Polythionine	HPLC-UV	0.5	87	At least 9 times	[85]
Gemfibrozil	Serum	β-CD	Spectrofluorometry	3 × 10^−3^	96.0–104.0	At least 50 times	[87]
SSRIs	Plasma	PAMAM	HPLC-UV	0.3–0.9	89.1–97.9	Up to 20 times	[78]
*Environmental Samples*							
Sulphonamide	Water	Porphyrin	HPLC-DAD	200	83.7–116.7	At least 8 times	[80]
Chlorophenols	Water	Poly (DVB-co-GMA)	HPLC-MS/MS	0.6–9.2	86.4–99.8	N.A.	[81]
PAHs	Water	Polystyrene	GC-FID	3 × 10^−3^–10 × 10^−3^	69.5–88.7	N.A.	[82]
PAHs	Water	Poly(pyrrole-co-aniline)	GC-FID	0.003–0.01	50.4–78.3	At least 20 times	[83]
Estrogens	Water	Triethylenetetramine	LC-MS/MS	0.15–1.5 × 10^−3^	88.5–105.6	N.A.	[88]
Malachite green	Water	SEP	UV Vis	0.2	96.3	At least 50 times	[79]
*Food Samples*							
Organophosphorus pesticides	Vegetables	Sporopollenin	GC-ECD	0.02–0.05	81.0–120.0	N.A.	[90]
Organophosphorus pesticides	Fruit, vegetables, water	Phenylethyl amine	GC-NPD	0.02–0.1	>80.4	At least 30 times	[91]
PAHs	Vegetable oils	Phytic acid	HPLC-DAD	0.06–0.15 ng g^−1^	85.6–102.3	At least 20 time	[86]
sildenafil citrate	Herbal supplementary products	Nanodiamond	HPLC-DAD	1.49	94.0–104.1	At least 10 times	[77]
*Agricultural Samples*							
Cytokinins	Plants	Silica	HPLC-MS/MS	93–120 × 10^3^	78.9–97.3	At least 30 times	[89]
